# Prevalence and Spectrum of *BRCA* Germline Variants in Central Italian High Risk or Familial Breast/Ovarian Cancer Patients: A Monocentric Study

**DOI:** 10.3390/genes11080925

**Published:** 2020-08-12

**Authors:** Jennifer Foglietta, Vienna Ludovini, Fortunato Bianconi, Lorenza Pistola, Maria Sole Reda, Antonella Al-Refaie, Francesca Romana Tofanetti, Annamaria Mosconi, Elisa Minenza, Paola Anastasi, Carmen Molica, Fabrizio Stracci, Fausto Roila

**Affiliations:** 1 Medical Oncology Division, S. Maria Hospital, 05100 Terni, Italy; j.foglietta@aospterni.it; 2 Medical Oncology Division, S. Maria della Misericordia Hospital, 06132 Perugia, Italy; lorenza.pistola@ospedale.perugia.it (L.P.); mariasolereda@inwind.it (M.S.R.); a_anto92@hotmail.it (A.A.-R.); francesca.tofanetti@ospedale.perugia.it (F.R.T.); annamaria.mosconi@ospedale.perugia.it (A.M.); elisa.minenza@ospedale.perugia.it (E.M.); paola.anastasi@ospedale.perugia.it (P.A.); carmen.molica@ospedale.perugia.it (C.M.); fausto.roila@ospedale.perugia.it (F.R.); 3 Umbria Cancer Registry, University of Perugia, 06129 Perugia, Italy; fortunato.bianconi@gmail.com; 4 Department of Experimental Medicine, Public Health Section, University of Perugia, 06129 Perugia, Italy; fabrizio.stracci@unipg.it

**Keywords:** *BRCA1/2* variant carrier, breast cancer, VUS, genetic testing, risk evaluation

## Abstract

Hereditary breast and ovarian cancers are mainly linked to variants in *BRCA1*/2 genes. Recently, data has shown that identification of *BRCA* variants has an immediate impact not only in cancer prevention but also in targeted therapeutic approaches. This prospective observational study characterized the overall germline *BRCA* variant and variant of uncertain significance (VUS) frequency and spectrum in individuals affected by breast (BC) or ovarian cancer (OC) and in healthy individuals at risk by sequencing the entire *BRCA* genes. Of the 363 probands analyzed, 50 (13.8%) were *BRCA1/2* mutated, 28 (7.7%) at *BRCA1* and 23 (6.3%) at *BRCA2* gene. The variant c.5266dupC p.(Gln1756Profs) was the most frequent alteration, representing 21.4% of the *BRCA1* variants and 12.0% of all variants identified. The variant c.6313delA p.(Ile2105Tyrfs) of *BRCA2* was the most frequent alteration observed in 6 patients. Interestingly, two new variants were identified in *BRCA2*. In addition, 25 different VUS were identified; two were reported for the first time in *BRCA1* and two in *BRCA2*. The number of triple-negative BCs was significantly higher in patients with the pathogenic *BRCA1/2*-variant (36.4%) than in *BRCA1/2* VUS (16.0%) and *BRCA1/2* wild-type patients (10.7%) (*p* < 0.001). Our study reveals that the overall frequency of *BRCA* germline variants in the selected high-risk Italian population is about 13.8%. We believe that our results could have significant implications for preventive strategies for unaffected *BRCA*-carriers and effective targeted treatments such as PARP inhibitors for patients with BC or OC.

## 1. Introduction

Breast cancer (BC) is the most common cancer and leading cause of cancer-related mortality among women worldwide. In Europe, approximately 500,000 women are diagnosed with BC annually, and in 2018, BC cases were responsible for a third of all cancer related deaths (about 130,000) [1]. Most women with breast or ovarian cancer (OC) have a sporadic rather than an inherited cancer. However, the majority of hereditary breast and ovarian cancers (HBOC) are due to highly penetrant germline *BRCA* variants, which are inherited in an autosomal-dominant fashion: breast cancer susceptibility gene 1 (*BRCA1*) or breast cancer susceptibility gene 2 (*BRCA2*). In these patients, there are frequently several generations of women affected with BC (often premenopausal) and, in some families, OC as well. The prevalence of *BRCA* variants varies based on a number of factors, including type of cancer and age at diagnosis. For individuals whose ethnicity is associated with higher variant frequency, particularly Ashkenazi Jews, any personal or family history of BC is sufficient to warrant consideration of *BRCA* testing. Aside from Ashkenazi Jews, founder variants have also been reported worldwide in populations from the Netherlands, Sweden, Hungary, Iceland, Italy, France, South Africa, Pakistan, Asia, and among French Canadians, Hispanics, and African Americans [2,3,4,5].

In a recent study, the incidences of BC and OC were reported to be 72% or 44% in *BRCA1* carriers and 69% or 17% in *BRCA2* carriers, respectively [6,7]. Other *BRCA*-associated malignancies such as prostate, male breast and pancreatic cancer may also be observed. Less commonly, BC is due to other hereditary syndromes, such as Li-Fraumeni and Cowden, which are associated with variants in the *TP53* and *PTEN* genes, respectively [8]. BC is the most prevalent cancer type and the first cause of death among women in Italy [9]. International guidelines, in cases of known variants in the family, early-onset or triple-negative cancers and multiple relatives with cancer, suggest referral for genetic counseling [10,11]. In recent years, poly(ADP-ribose) polymerase (PARP) inhibitors have been developed that target *BRCA* pathogenic variants in various cancer types including breast and ovarian cancers [12]. Thus, the detection of *BRCA* variants has a relevant impact both in cancer prevention and in targeted treatment. Typically, variant screening has been performed among affected women, selected on the basis of young age at diagnosis or family cancer history. The aim of this study is to determine the overall germline *BRCA* variant frequency and spectrum in healthy Italian individuals at risk or affected by BC or OC by molecular genetic analysis of regions of *BRCA1* and *BRCA2* genes.

## 2. Materials and Methods

### 2.1. Patients and Samples

Individuals referring to genetic counseling at the Medical Oncology Division of the S. Maria della Misericordia Hospital (Perugia-Italy) in the years 2010–2016 at risk or with a history of BC or OC were included in the study. This cohort of 363 women/men was selected according to the Italian Medical Oncology Association (AIOM) guidelines [13] based on age at BC/OC onset, number of cancer cases in I- and II degree relatives, and pathological characteristics of BC. Several genetic risk assessment methods are available to estimate the probability of *BRCA* variant in individuals in order to select them for molecular diagnosis [14]. Genetic testing was performed on all individuals >18 years old selected according to the AIOM guidelines and these criteria do not differ from other jurisdictions in Italy.

- Knowledge of pathogenetic mutation in the family
- Males affected by breast cancer
- Women with breast and ovarian cancer
- Women affected by breast cancer <36 years old
- Women affected by triple negative breast cancer <60 years old
- Women with bilateral breast cancer <50 years old
- Women with breast cancer <50 years old AND first degree familiarity of:
  1. Breast cancer <50 years old
  2. No-mucinous, no-border line ovarian cancer (all ages)
  3. Bilateral breast cancer
  4. Male breast cancer
  5. Pancreatic cancer
  6. Prostate cancer

We chose, however, to utilize *BRCA*PRO software that is based on Bayes’ theorem; this requires data on all first, second and third degree relatives of the family proband and incorporates as prior probabilities incidence rates in the US population, allele variant frequencies and penetrances estimated from studies in families with several BC or OC cases [15,16,17]. For unaffected individuals we utilized the Cuzick–Tyrer model that, developed for the International Breast Intervention Study (IBIS-1), incorporates the assessment of additional hereditary factors, body mass index, menopausal status and hormone replacement therapy use [18]. We considered it suitable for genetic testing of *BRCA* variant individuals with an estimated life-time risk of disease ≥10%. The study was conducted in accordance with Good Clinical Practice and the ethical principles of the Declaration of Helsinki and approved by the S. Maria della Misericordia Ethics Committee (CE, protocol 2207/2010). We obtained written informed consent from all participants. Clinical data such as age at diagnosis, hystotype, grading, stage, tumor invasiveness, and receptor status were gathered.

St Gallen guidelines were used to classify BC subtype, based on receptor status [19]. Data about a second BC and/or OC or other malignancies and the family cancer history in I and II degree relatives were also collected.

### 2.2. BRCA1/2 Analysis

Ten milliliters of whole blood mixed with EDTA were collected from each patient. Genomic DNA was extracted from blood using the QIAamp DNA mini kit (Qiagen, Hilden, Germany) and quantified using the Qubit dsDNA BR Assay Kit (ThermoFisher Scientific, M0, Italy). All 23 coding exons of *BRCA1* (exons 2 to 24) and 26 coding exons of *BRCA2* (exons 2 to 27) were amplified in 33 and 46 amplicons, respectively. The primers were designed to cover all coding exons and adjacent 20 base pair introns. The amplified DNA fragments were sequenced using the BigDyeTerminator v.3.1 cycle sequencing kit (Thermo Fisher Scientific) on a 3500 Genetic analyzer (Applied Biosystems, Foster City, CA, USA). Sequencing chromatograms were analyzed for variant detection using Seqscape software v.2.7 (Applied Biosystems, Foster City, CA, USA). In all cases, samples harboring variants were re-amplified and re-sequenced using the same experimental conditions. All sequences were compared with the *BRCA1* (NM_007294.3) and *BRCA2* (NM_000059.3) reference sequences for variant detection. To identify gross deletions/insertions not detectable by sequencing on the *BRCA1*/2 genes, we performed the Multiplex Ligation-dependent Probe Amplification (MLPA) using the SALSA P002 *BRCA1* and SALSA P045 *BRCA2* MLPA probe mix assays (MRC-Holland, Amsterdam, The Netherlands) according to the manufacturer’s instructions. Coffalyser V9.4 software (MRC-Holland, Amsterdam, The Netherlands) was used to analyze MLPA results.

### 2.3. Variant Classification

According to the IARC recommendations [20], we classified genetic variants identified into five classes. To annotate *BRCA1/2* variants we used: databases such as Breast Cancer Information Core (BIC) [21], *BRCA* Share (formerly Universal Variant Database) [22], Leiden Open Variation Database (LOVD) [23], ClinVar-NCBI Database, and American College of Medical Genetics (ACMG) guidelines [24].

Variants not found in these databases were classified on the basis of their characteristics.

All variants with conflicting interpretation results by ClinVar-NCBI Database were considered as VUSs. The classification of variants initially considered as VUS was subjected to regular updates, by reviewing the literature and publicly available databases to the best of our knowledge, and modified accordingly. Frameshift and nonsense VUS leading to a premature stop codon were considered likely-pathogenic-class4 and classified in accordance with the ACMG guidelines. All variants were reported according to Human Genome Variation Society nomenclature [25] according to ENIGMA (Evidence-based Network for the Interpretation of Germline Mutant Alleles) consortium rules for variant classification to obtain the most recent information on variant reclassifications.

### 2.4. Data Collection and Statistical Analysis

Data were collected using a management system that is integrated with the Umbria Cancer Registry application system [26].

Descriptive statistics of patients’ characteristics and sequencing results were presented as median and range for continuous data and as natural frequencies and percentages for categorical data. Pearson Chi-square test or an appropriate Fisher Exact test were used to compare tabular proportions. All data analyses were performed using R software version 3.4.2 (R Foundation for Statistical Computing, Vienna, Austria).

### 2.5. Immunohistochemistry Analysis of Breast Tumor Samples

Tumor immunohistochemical (IHC) analysis was performed for estrogen receptor (ER) (clone 1D5 diluted 1:15), progesterone receptor (PgR) (clone 1A6 diluted 1:15), and Ki-67 (clone MIB1 diluted 1:15) using the automated platform Bond III (Leica Biosystem, MI, Italy). IHC analysis for evaluation of human epidermal growth factor receptor 2 (*HER2*) status was performed using the HercepTest^TM^ kit (Dako, Glostrup, Denmark) with an automated system (Autostainer Link 48, Dako) according to the manufacturer’s instructions. *HER2* status was defined as negative (HercepTest scores of 0 or 1 +), doubtful (2 + score), and positive (3 ± score). To confirm *HER2* status when IHC results were doubtful, we used Fluorescence in-situ hybridization test using a HER2 FISH PharmDx^TM^ kit (Dako Glostrup, Denmark), and gene amplification was recorded when the HER2/centromeric probe for chromosome 17 signal ratio was ≥2.0.

## 3. Results

### 3.1. Patient Characteristics

This prospective observational study included 363 Central Italian individuals: 263 (72.4%) with BC (median age 46 years), 16 (4.4%) with other tumors, and 84 (23.1%) with no tumor. Of the 263 BC patients, 217 (82.5%) had a first BC, 44 (16.7%) a second BC and 2 (0.8%) had subsequent three BC. Among the 10 patients with OC, 3 had initial OC and 7 had a second OC after BC. The *BRCA2* pathogenic variants were significantly prevalent in patients with initial BC (*p* = 0.006, Fisher Exact test) while *BRCA1* pathogenic variants were significantly present in patients with OC (*p* < 0.001, Fisher Exact test). BC and OC patient tumor characteristics are summarized in Table 1. The majority of individuals genotyped with no a priori data on familial variant 269/363 (74.1%) were tested because of personal history of cancer while 94/363 (25.9%) were referred for oncogenetic counselling and genotyping because of a family history suggestive of inherited predisposition to cancer.

### 3.2. BRCA Variants and Patient Characteristics

A total of 363 oncogenetic genotyping results were performed in the present study, 351 in females (97.7%) and 12 (3.3%) in males. Overall, 50/363 (13.8%) genotyping individuals carried one pathogenic/likely pathogenic variant in either *BRCA* gene, including 28 (7.7%) pathogenic/likely pathogenic *BRCA1* variants and 23 (6.3%) pathogenic/likely pathogenic *BRCA2* variants (Table 2A). One patient had two variants in both *BRCA1* and *BRCA2* genes (sample ID 606, Table 2A). Thirteen of 50 (26.0%) variants found were carried in people with no history of cancer and 38/50 variants (76.0%) were detected in patients affected by BC. Of the BC *BRCA*-mutated patients, 21 (56.7%) were affected by a variant of *BRCA1* and 17 (45.3%) by a *BRCA2* variant. Of 13 women or men without personal history of cancer, 7 (53.8%) were affected by variants of *BRCA1* and 6 (46.2%) by variants of *BRCA2.* On the whole, the majority of *BRCA* pathogenic variants were reported to be in exon 11 for both genes: 10 (43.5%) variants in exon 11 of *BRCA1* and 13 (56.5%) of *BRCA2* gene, respectively. All detected pathogenic/likely pathogenic variants with the exception of three in splice sites of *BRCA2* gene and three variants missense of *BRCA1* gene, the cause being either termination or a frameshift in *BRCA* proteins. Five *BRCA*-variant carriers (17.9%) were affected from both BC and OC. Of seven patients presented with bilateral BC (14.6%), three *BRCA1* and four *BRCA2* pathogenic variants were found.

### 3.3. Cohort Spectrum and Variant Detection Rate

Table 2A lists the pathogenic/likely-pathogenic variants detected in the *BRCA1* and *BRCA2* genes, and Table 2B shows the *BRCA1* and *BRCA2* VUS variants as well as their frequencies. We found 14 different pathogenic/likely-pathogenic variants in *BRCA1* gene and 16 in *BRCA2* gene. Overall, of the 30 pathogenic/likely-pathogenic variants, 2 (6.6%) were novel variants in exon 17 of *BRCA2* (c.7828_7834delGTGGATC p.(Val2610fs); c.7852_7862delATTTGGGTTTA, p.(Ile2618fs)) not previously reported in BIC, LOVD, ClinVar-NCBI Database, *BRCA*-Share or any published literature. Besides the detrimental variant detected, 9 and 16 VUS were identified in the *BRCA1* and *BRCA2* genes, respectively. Of these 25 *BRCA1/2* VUS, 2 are reported here for the first time in *BRCA1* (c.4986 + 47A > G (IVS16 + 47A > G) in exon 16; c.5407-72delAAAA (IVS22-72delAAAA) in exon 23) and 2 in *BRCA2* (c.4504C > A p.(Gln1502Lys) in exon 11; c.7618-11delATTTT (IVS15-11delATTTT) in exon 16). The most frequent VUS variant detected in exon 11 of *BRCA2* c.5972C > T p.(Ala1991Val) was observed in five patients. Seven women presented at the same time a VUS and a pathogenic variant, three patients with VUS resulted affected by both OC and BC and six patients had bilateral BC.

### 3.4. Recurrent Pathogenic/Likely-Pathogenic BRCA1/2 Variants

Of the 30 distinct pathogenic/likely-pathogenic *BRCA* variants in our patient cohort, 23 were observed only once; 5 in *BRCA1* and 2 in *BRCA2* variants were detected in at least two or more. These seven variants were detected in 23.3% of all patients with pathogenic *BRCA* variant. The most frequent pathogenic variant detected in *BRCA1* c.5266dupC p.(Gln1756Profs) exon 20 and *BRCA2* c.6313delA p.(Ile2105Tyrfs) exon 11, was observed in six patients, respectively (Table 2A).

### 3.5. Characteristics of Breast Cancer in BRCA Carrier Patients

**Figure 1:**
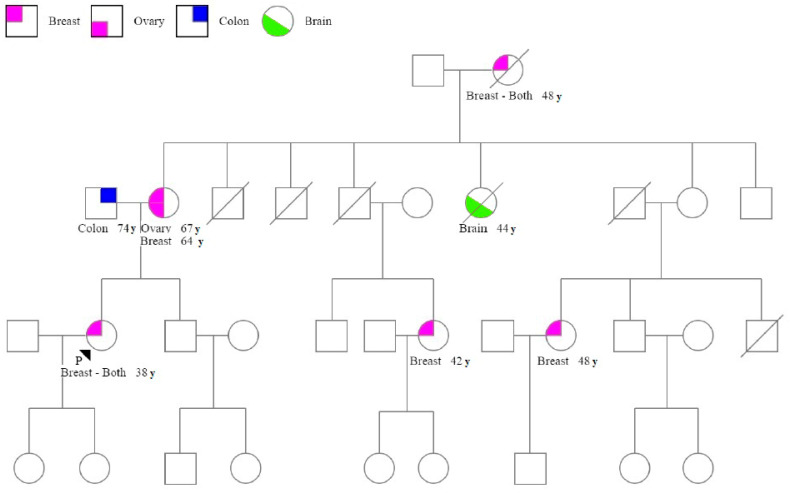
Pedigree of patient ID 48 with c.6313delA p.(Ile2105Tyrfs) pathogenic variant in the *BRCA2* gene. The proband is indicated by a black arrow. Cancer Type and age at cancer diagnosis is indicated in the legend. Symbols: squares = males, circles = females; quadrant shading = cancer affected; slash through square or circle = deceased.

Table 3 describes the characteristics of BC in patients with pathogenic/likely pathogenic *BRCA1/2* variants in comparison with patients with *BRCA1/2*-VUS and without *BRCA1/2* variants. Median age of the 33 patients with pathogenic/likely pathogenic *BRCA1/2* variant was 46 years (range 27–65). The most frequent histology was ductal (*n* = 21, 63.6%), followed by lobular in seven (21.2%) patients and other invasive histotypes in five (15.2%) (*p* = 0.005, Fisher Exact test). VUS BRCA2 variants were observed with significant differences in patients with invasive tumor with respect to patients with in situ carcinoma (70% vs. 30% respectively, *p* = 0,014 Fisher Exact test). According to surrogate definitions of intrinsic subtypes of breast cancer, 36.4% of tumors were classified as triple negative, 45.5% as luminal a-like breast cancer and 3.0% as luminal b-like. The number of triple-negative BCs (TNBCs) was significantly higher in patients with pathogenic *BRCA1/2*-variant (36.4%) than in *BRCA1/2* VUS (16.0%) and *BRCA1/2* wild type patients (10.7%) (*p* < 0.001, Fisher Exact test). No enriched HER-2 was found in patients with pathogenic *BRCA1/2* variant. In situ carcinoma was significantly observed in 32% of patients with *BRCA1/2* VUS with respect to the 11.2% of patients without *BRCA1/2* variant (*p* = 0.005, Fisher Exact test). The pathogenic *BRCA1/2* variant was observed more often in patients with high Ki67 (81.8%) than in those with *BRCA1/2*-VUS (44.0%) and in those without *BRCA1/2* variant (52.7%) (*p* = 0.008, Fisher Exact test). No significant differences were detected in terms of median age, stage, grading, and exitus. An example is shown in Figure 1: the family members of the proband harboring the pathogenic variant c.6313delA in the *BRCA2* gene. As shown in the pedigree, the proband diagnosed with bilateral breast cancer at the age of 38 carried the pathogenic variant in *BRCA2*. She had a first-degree relative with both ovarian and breast cancer and a second-degree relative with bilateral breast cancer. Estimated variant probability for *BRCA1/2* before genetic testing was 26.6% by Myriad and 18.4% by *BRCA*PRO. Genetic testing was performed on her two cousins with breast cancer who carried a *BRCA2* gene with the same pathogenic variant. Her two daughters without breast cancer had the same pathogenic variant.

### 3.6. Characteristics of Breast Cancer in Patients with VUS

**Figure 2:**
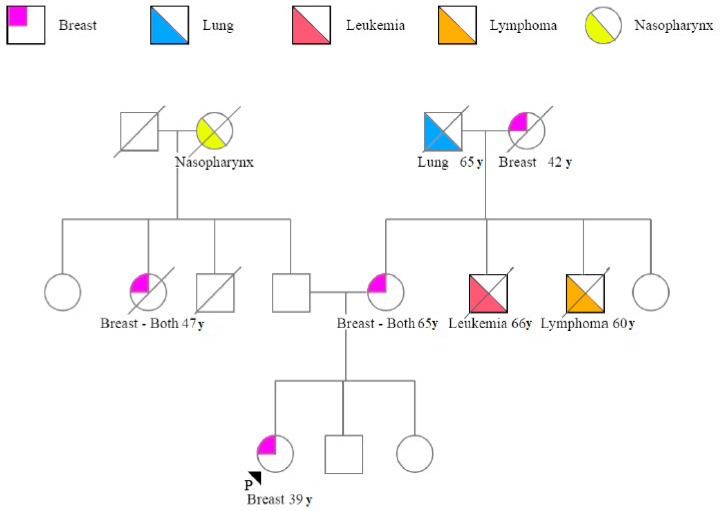
Pedigree of patient ID 886 with c.4928T>C, p.(Val1643Ala) Unclassified variant in *BRCA2* gene. The proband is indicated by a black arrow. Cancer Type and age at cancer diagnosis is indicated in the legend. Symbols: squares = males, circles = females; quadrant shading = cancer affected; slash through square or circle = deceased.

Mean age of the 25 patients with *BRCA1/2* VUS was 48 years (range 34–68) and the most frequent histology was ductal (40.0%), followed by lobular 4.0% with other invasive hystotypes 24.0%. Grade 1 was detected in 24.0% of breast cancer, G2 in 36.0%, G3 in 24.0%; information about grading was missing in 16.0% of cases. According to surrogate definitions of intrinsic subtypes of breast cancer [20], 16.0% of tumors were classified as triple negative, 28.0% as luminal a-like breast cancer and 12.0% as luminal b-like. No enriched HER-2 was found in patients with *BRCA1/2* VUS. Figure 2 shows the pedigree of a family with VUS. The proband harboring the c.4928T > C variant in the *BRCA2* gene was diagnosed with breast cancer at the age of 39; her mother suffered from bilateral BC and carried the same VUS. Her aunt (mother’s sister) died of breast cancer as did her grandmother (*BRCA* test not performed). This VUS seems representative of the hereditary factor of BC due to the frequency of cases with bilateral breast cancer and the onset in youth in three relatives present in the maternal line (mother, aunt and maternal grandmother). 

## 4. Discussion

This is a Central Italian study evaluating the prevalence and spectrum of *BRCA1/2* variants. We focused our study on variant detection rates and genetic characteristics associated with specific selection criteria for *BRCA1/2* testing in high-risk families and patients affected by breast cancer, whereas other authors evaluated clinical implications and strategy of surveillance of women at high risk. Thirteen percent of the individuals evaluated were carriers of a pathogenic variant, according to the range shown in other countries [27,28,29,30], excluding Ashkenazi Jewish ancestry in which founder variants were prevalent [31]. The incidence of *BRCA1* and *BRCA2* variants was 7.7% and 6.3%, respectively. According to the literature, we report an incidence of TNBC in *BRCA*-carriers (36.4%) about 2-fold higher than that found in sporadic breast cancer. TNBC has been reported to account for 12–24% of all BCs and is associated with an hereditary disease cause [32,33]. Approximately 70% of BCs found in *BRCA1* variant carriers and up to 23% of BCs in *BRCA2* carriers are triple-negative [34]. Therefore, according to national and international guidelines, women with TNBC diagnosed at an age ≤50–60 years, irrespective of a positive cancer family history, are eligible for germline *BRCA* testing [11,12,13]. As reported in the literature [35,36], *BRCA*-mutated BC patients showed a significant number of triple-negative cancers (*p* < 0.001) and higher Ki-67 expression (*p* = 0.008) than in other patients (Table 3), which represents the higher aggressiveness of the disease. *BRCA1* pathogenic/likely pathogenic variants reported in our study were higher than *BRCA2* variants (54.9% and 45.0%, respectively). More than 2000 different variants have been identified in *BRCA1/2* genes and in some populations, founder variants are the most prevalent ones. For example, up to 2.5% of the general Ashkenazi Jewish population will harbor variants in *BRCA1* c.68_69delAG (also known as 185delAG), c.5266dupC (also known as 5382insC) or *BRCA2* c.5946delT (also known as 6174delT) [37].

We observed 30 distinct pathogenic/likely pathogenic *BRCA* variants (14 in *BRCA1* and 16 in *BRCA2*) and while 23 were observed only once, 5 in *BRCA1* and 2 in *BRCA2* variants were detected at least two or more times. These seven variants were detected in 23.3% of all the patients with pathogenic *BRCA* variant and almost all of them were observed in exon 20 of *BRCA1* and exon 11 of *BRCA2.* It is important to screen individual populations and ethnic groups to evaluate the true prevalence of *BRCA* germline variants [38], as the frequency and type of *BRCA* variants vary significantly depending on ethnicity and race. To our knowledge, our *BRCA* study on an Italian population (breast/ovarian cancer patients and healthy population) showed that when several recurrent pathogenic variants are detected, these may be considered as founder variants for this population. If confirmed by further studies, this could have significant implications for preventive population screening and targeted treatments with PARP inhibitors. In our cohort, the *BRCA1* c.5266dupC (also known as 5382dupC), considered the founder variant of North-Eastern European origin, was the most frequent, representing 23% of *BRCA1* variant carriers, as reported in a previous Italian study [39].

In our study, of the 30 pathogenic-likely pathogenic variants observed, 2 (6.6%) are novel and it will be necessary to evaluate their level of penetration in carrier families.

Moreover, different *BRCA* variants lead to protein alterations that could have a different impact on the risk of developing tumors in *BRCA* variant carriers [40].

If a high risk *BRCA* variant should be detected, it is important to perform genetic counselling to guide patients and their families regarding risk reduction options and treatment. In our study, we have reported a list of the VUS identified (mostly missense variants) and we note a lack of consensus about their biological/clinical significance among the different databases. Based on the frequency or the co-occurrence of pathogenic variants of these VUS, found in the small number of cases tested in our center, it was not possible to classify these variants. Even though clinician’s decisions cannot be made based on VUS, some of our findings are worthy of attention and deserve further investigation. This is the case, for example, of the young patient (39 years old) with the variant c.4928T > C reported in *BRCA2* (Figure 2). Segregation analysis and functional studies should be further performed in this family due to the absence of consensus among databases. Moreover, other breast/ovarian cancer predisposition genes (already present in commercial panels) should also be investigated by next-generation sequencing.

A strength of our study is that it considers not only the affected individuals but also healthy people considered at risk on the basis of the Cuzick–Tyrer program (life-time risk cut off: 10%). Indeed, studies evaluating only patients affected might lead to an overestimate of probability of detecting a variant.

A possible limitation of our study is the selection of individuals for testing. Women should probably not be selected for *BRCA* testing using only protocols based on risk evaluation tools and strict probability thresholds. Furthermore, there are several different tools to evaluate *BRCA* risk, and we do not know which is best. Of course, programs with a proactive approach of genetic counseling probably need to enforce rigid selection criteria based on probability threshold in order to contain costs and safeguard their feasibility and ethical sustainability. Besides the variant risk, a woman’s personal motivation and the potential utility of test results for the family should be considered. Another limitation of our study is the absence of segregation analysis within family members that could facilitate follow up of people at high risk of disease and their relatives.

Notwithstanding these limitations, our study provides the identification of patients with heterozygous variants of both *BRCA1* and *BRCA2*, along with individuals carrying one variant and a VUS, underlining the necessity of complete *BRCA1*/2 testing, which should be offered to all eligible individuals.

The increase of genetic testing leads to the probability of having an non-informative result or VUS. For the management of VUS, it is important to evaluate family history, clinical factors and functional studies on *BRCA* protein.

Because this information can be confusing and anxiety-provoking to patients, international collaborative efforts are strongly encouraged to ensure that data pertaining to VUS are publicly available.

## 5. Conclusions

Our study reveals that the overall frequency of *BRCA* germline variants in the selected high-risk central Italian population (BC or OC patients and healthy individuals with elevated risk of hereditary BC or OC) is about 13.8%. Further, several recurrent pathogenic variants detected could be considered as founder variants, if confirmed by further studies. We believe that our results could have significant implications for preventive strategies for unaffected *BRCA*-carriers and effective targeted treatments such as PARP inhibitors for patients with BC or OC.

## Figures and Tables

**Table 1:** Population Characteristics.

			*BRCA1*			*BRCA2*			*BRCA1/2*		
			Pathogenic/Likely Pathogenic Variants	VUS	No Pathogenic Variants	Pathogenic/Likely Pathogenic Variants	VUS	No Pathogenic Variants	Pathogenic/Likely Pathogenic Variants	VUS	No Pathogenic Variants
*** Overall Central Italian individuals (N. %)		363 (100.0)	28 (7.7)	9 (2.5)	326 (89.8)	23 (6.3)	21 (5.8)	319 (87.9)	50 ** (13.8)	28 (7.7)	285 (78.5)
**Age at diagnosis, years**	**Median** **Range (Min-Max)**	47 (19–84)	49 (22–69)	54 (37–74)	47 (19–84)	48 (19–84)	50 (27–72)	47 (19–81)	48 (19–84)	51 (27–74)	47 (19–81)
	*p*-value *		0.165			0.444			0.09		
		N (%)									
**Sex**											
	**Female**	351 (97.7)	27 (96.4)	8 (88.9)	316 (96.9)	22 (95.7)	21 (100.0)	308 (96.6)	48 (96.0)	27 (96.4)	276 (96.8)
	**Male**	12 (3.3)	1 (3.6)	1 (11.1)	10 (3.1)	1 (4.3)	0 (0.0)	11 (3.4)	2 (4.0)	1 (3.6)	9 (3.2)
	*p*-value *		0.411			0.665			0.961		
**Tumor Type**											
**BC**											
	**First BC**	217 (59.9)	15 (53.6)	5 (55.6)	197 (60.4)	11 (47.8)	13 (61.9)	193 (60.5)	25 (50.0)	14 (57.1)	176 (61.8)
	**Second BC**	44 (12.1)	4 (14.3)	2 (22.2)	38 (11.7)	3 (13.0)	7 (33.3)	34 (10.7)	7 (14.0)	9 (32.2)	28 (9.8)
	**Third BC**	2 (0.5)	0 (0.0)	0 (0.0)	2 (0.6)	1 (4.4)	0 (0.0)	1 (0.3)	1 (2.0)	0 (0.0)	1 (0.4)
	**Other tumors**	16 (4.4)	2 (7.1)	1 (11.1)	13 (4.0)	2 (8.7)	1 (4.8)	13 (4)	4 (8.0)	2 (7.1)	10 (3.5)
	**No tumors**	84 (23.1)	7 (25)	1 (11.1)	76 (23.3)	6 (26.1)	0 (0.0)	78 (24.5)	13 (26.0)	1 (3.6)	70 (24.5)
	*p*-value *		0.898			**0.006**			**0.006**		
**OC**											
	**First OC**	3 (0.8)	2 (7.1)	0 (0.0)	1 (0.3)	0 (0.0)	0 (0.0)	3 (0.9)	2 (4.0)	0 (0.0)	1 (0.3)
	**Both BC and OC**	7 (1.9)	5 (17.9)	0 (0.0)	2 (0.6)	0 (0.0)	1 (4.8)	6 (1.9)	5 (10.0)	1 (3.6)	1 (0.3)
	**No**	269 (96.4)	21 (75.0)	9 (100.0)	323 (99.1)	23 (100.0)	20 (95.2)	310 (97.2)	43 (86.0)	27 (96.4)	283 (99.3)
	*p*-value *		**<0.001**			0.779			**<0.001**		

Abbreviations: BC, breast cancer; OC, ovarian cancer; VUS, variant of uncertain significance. * Pearson Chi-square test or the Fisher Exact test, as appropriate. ** One patients possess the pathogenic variants of the both *BRCA1* and *BRCA2* genes simultaneously (ID 606). *** the individuals were all Caucasians.

**Table 2:** (A) List of *BRCA1* and *BRCA2* pathogenic/likely pathogenic variants detected in 50 Central Italian individuals. (B) List of *BRCA1* and *BRCA2* Variants of Uncertain Significance (VUS) variants detected in 33 Central Italian individuals *.

Table 2 (A) List of *BRCA1* and *BRCA2* Pathogenic/Likely-Pathogenic Variants Detected on 50 Central Italian Individuals									
Sample ID	Gene	Exon/Intron	HGVS cDNA (*BRCA1* NM_007294.3) (*BRCA2* NM_000059.3)	HGVS Protein	Variant Type	IARC Classification	ClinVar	*BRCA* Share- BIC-LOVD	N.
66,101	*BRCA1*	2	c.68_69delAG	p.(Glu23Valfs)	Frameshift deletion	Class-5	Pathogenic	Pathogenic	2
315	*BRCA1*	3	c.116G > A	p.(Cys39Tyr)	Missense	Class-5	Pathogenic	Pathogenic	1
909	*BRCA1*	5	c.181T > G	p.(Cys61Gly)	Missense	Class-5	Pathogenic	Pathogenic	1
403	*BRCA1*	11	c.1999C > T	p.(Gln667Ter)	Nonsense	Class-5	Pathogenic	Pathogenic	1
833	*BRCA1*	11	c.3228_3229delAG	p.(Gly1077Alafs)	Frameshift deletion	Class-5	Pathogenic	Pathogenic	1
265,287,471,524	*BRCA1*	11	c.2406_2409delGAGT	p.(Gln804Valfs)	Frameshift deletion	Class-5	Pathogenic	Pathogenic	4
475,606,1341	*BRCA1*	11	c.3326-3329delAAAA	p.(Lys1109Serfs)	Frameshift deletion	Class-5	Pathogenic	Pathogenic	3
223	*BRCA1*	11	c.3599_3600delAG	p.(Gln1200Argfs)	Frameshift deletion	Class-5	Pathogenic	Pathogenic	1
443	*BRCA1*	12	c.4117G > T	p.(Glu1373Ter)	Nonsense	Class-5	Pathogenic	Pathogenic	1
161	*BRCA1*	16	c.4964_4982del19	p.(Ser1655Tyrfs)	Frameshift deletion	Class-5	Pathogenic	Pathogenic	1
270,300,358,1011	*BRCA1*	17	c.5062_5064delGTT	p.(Val1688del)	Inframe deletion	Class-5	Pathogenic	Pathogenic	4
50	*BRCA1*	18	c.5096G > A	p.(Arg1699Gln)	Missense	Class-5	Pathogenic	Pathogenic	1
47,150,746,938,943,609	*BRCA1*	20	c.5266dupC	p.(Gln1756Profs)	Frameshift insertion	Class-5	Pathogenic	Pathogenic	6
932	*BRCA1*	23	c.5445G > A	p.(Trp1815Ter)	Nonsense	Class-5	Pathogenic	Pathogenic	1
616	*BRCA2*	2	c.67 + 1G > A	-	Splicing	Class-5	Pathogenic	Pathogenic	1
606	*BRCA2*	8	c.632 − 2A > G	-	Splicing	Class-5	Pathogenic	Pathogenic	1
289	*BRCA2*	8	c.658_659delGT	p.(Val220Ilefs)	Frameshift deletion	Class-5	Pathogenic	Pathogenic	1
426	*BRCA2*	11	c.3919delG	p.(Glu1307Lysfs)	Frameshift deletion	Class-5	Pathogenic	Pathogenic	1
352	*BRCA2*	11	c.4284dupT	p.(Gln1429Serfs)	Frameshift deletion	Class-5	Pathogenic	Pathogenic	1
959	*BRCA2*	11	c.5645C > A	p.(Ser1882Ter)	Nonsense	Class-5	Pathogenic	Pathogenic	1
865,946,1004	*BRCA2*	11	c.5722_5723delCT	p.(Leu1908Argfs)	Frameshift deletion	Class-5	Pathogenic	Pathogenic	3
424	*BRCA2*	11	c.6039delA	p.(Val2014Tyrfs)	Frameshift deletion	Class-5	Pathogenic	Pathogenic	1
48,78,291,564,614,615	*BRCA2*	11	c.6313delA	p.(Ile2105Tyrfs)	Frameshift deletion	Class-5	Pathogenic	Pathogenic	6
618	*BRCA2*	17	c.7828_7834delGTGGATC	p.(Val2610fs)	Frameshift deletion	Class-4	-	-	1
367	*BRCA2*	17	c.7852_7862delATTTGGGTTTA	p.(Ile2618fs)	Frameshift deletion	Class-4	-	-	1
260	*BRCA2*	18	c.8174G > A	p.(Trp2725Ter)	Nonsense	Class-5	Pathogenic	Pathogenic	1
393	*BRCA2*	19	c.8487 + 1G > A	-	Splicing	Class-5	Pathogenic	UV/Pathogenic	1
295	*BRCA2*	20	c.8537_8538delAG	p.(Glu2846Glyfs)	Frameshift deletion	Class-5	Pathogenic	Pathogenic	1
640	*BRCA2*	22	c.8878C > T	p.(Gln2960Ter)	Nonsense	Class-5	Pathogenic	Pathogenic	1
571	*BRCA2*	22	c.8930delA	p.(Tyr2977Phefs)	Frameshift deletion	Class-5	Pathogenic	Pathogenic	1
**Table 2 (B) List of *BRCA1* and *BRCA2* Variant of Uncertain Significance (VUS) Variants Detected in 33 Central Italian Individuals**									
**Sample ID**	**Gene**	**Exon/Intron**	**HGVS cDNA** **(*BRCA1* NM_007294.3)** **(*BRCA2* NM_000059.3)**	**HGVS Protein**	**Variant Type**	**IARC Classification**	**Clin Var**	***BRCA* Share-BIC-LOVD**	**N.**
879	*BRCA1*	2	c.-77delTGT (IVS0-77delTGT)	-	Intron	Class-3	-	-	1
733	*BRCA1*	7	c.335A > G	p.(Asn112Ser)	missense	Class-3	-	VUS	1
632	*BRCA1*	11	c.734A > T	p.(Asp245Val)	missense	Class-3	VUS	VUS	1
635	*BRCA1*	11	c.3711A > G	p.(Ile1237Met)	missense	Class-3	VUS	VUS	1
303	*BRCA1*	12	c.4132G > A	p.(Val1378Ile)	missense	Class-3	VUS	VUS	1
1013	*BRCA1*	16	c.4986 + 47A > G (IVS16+47A > G)	-	Intron	Class-3	-	-	1
635	*BRCA1*	16	c.4843G > A	p.(Ala1615Thr)	missense	Class-3	VUS	VUS	1
272,478	*BRCA1*	20	c.5277 + 60_5277 + 61insGTATTCCACTCC	-	Intron	Class-3	VUS	Benign/VUS	2
1012	*BRCA1*	23	c.5407-72delAAAA	-	Intron	Class-3	-	-	1
527	*BRCA2*	2	c.67 + 62T > G (IVS2+62T>G)	-	Intron	Class-3	VUS	Benign/VUS	1
553	*BRCA2*	10	c.1181A > C	p.(Glu394Ala)	missense	Class-3	VUS	VUS	1
886,930	*BRCA2*	11	c.4928T > C	p.(Val1643Ala)	missense	Class-3	VUS	VUS	2
633	*BRCA2*	11	c.4504C > A	p.(Gln1502Lys)	missense	Class-3	-	-	1
399,532,558,635,679	*BRCA2*	11	c.5972C > T	p.(Ala1991Val)	missense	Class-3	VUS	VUS	5
212,309	*BRCA2*	11	c.6131G > C	p.(Gly2044Ala)	missense	Class-3	VUS	VUS	2
423	*BRCA2*	11	c.6441C > G	p.His2147Gln)	missense	Class-3	VUS	VUS	1
259,296	*BRCA2*	11	c.6461A > C	p.(Tyr2154Ser)	missense	Class-3	VUS	VUS	2
752	*BRCA2*	11	c.6641C > T	p.(Thr2214Ile)	missense	Class-3	VUS	VUS	1
518	*BRCA2*	15	c.7505G > A	p.(Arg2502His)	missense	Class-3	VUS	VUS	1
367	*BRCA2*	16	c.7618-11delATTTT	-	Intron	Class-3	-	-	1
571	*BRCA2*	25	c.9275A > G	p.(Tyr3092Cys)	missense	Class-3	VUS	VUS	1
1012	*BRCA2*	25	c.9501 + 3A > T	-	Intron	Class-3	VUS	VUS	1
786	*BRCA2*	26	c.9648 + 84G > A	-	Intron	Class-3	VUS	Likely Benign/VUS	1
64	*BRCA2*	27	c.10024G > A	p.(Glu3342Lys)	missense	Class-3	VUS	VUS	1
1016	*BRCA2*	27	c.10095delinsGAATTATATCT	p.(Ser3366fs)	Frameshift deletion	Class-3	VUS	Benign/VUS	1

Abbreviations: HGVS, Human Genome Variation Society; cDNA, coding DNA; IARC, International Agency for Research on Cancer; BIC, Breast Cancer Variant Data Base; *LOVD,* Leiden Open Variation Database; VUS, Variant of Uncertain Significance. *** the individuals were all Caucasians.

**Table 3:** Clinical features and *BRCA* status in BC.

			*BRCA1*			*BRCA2*			*BRCA1/2*		
			Variants	VUS	No Pathogenic Variants	Variants	VUS	No Pathogenic Variants	Variants	VUS	No Pathogenic Variants
*** Overall Central Italian individuals (N. %)		263 (100)	19 (7.2)	7 (2.7)	237 (90.1)	15 (5.7)	20 (7.6)	228 (86.7)	33 ** (12.6)	25 (9.5)	205 (77.9)
Age at diagnosis, years	Median Range(Min-Max)	46 (27–77)	47 (31–63)	47 (37–58)	46 (27–77)	44 (27–65)	50 (34–68)	46 (27–77)	46 (27–65)	48 (34–68)	46 (27–77)
	*p*-value *		0.784			0.194			0.169		
Histology											
	In situ carcinoma	31 (11.8)	0 (0.0)	2 (28.6)	29 (12.2)	0 (0.0)	6 (30.0)	25 (11.0)	0 (0.0)	8 (32.0)	23 (11.2)
	Invasive ductal carcinoma	152 (57.8)	13 (68.4)	2 (28.6)	137 (57.8)	9 (60.0)	8 (40.0)	135 (59.2)	21 (63.6)	10 (40.0)	121 (59.0)
	Invasive lobular carcinoma	33 (12.6)	3 (15.8)	1 (14.2)	29 (12.2)	4 (26.7)	1 (5.0)	28 (12.3)	7 (21.2)	1 (4.0)	25 (12.2)
	Other invasive hystotypes	47 (17.8)	3 (15.8)	2 (28.6)	42 (17.8)	2 (13.3)	5 (25.0)	40 (17.5)	5 (15.2)	6 (24.0)	36 (17.6)
	*p*-value *		0.418			**0.047**			**0.005**		
Grading											
	Well-differentiated	21 (8.0)	4 (21,1)	2 (28.6)	35 (14.8)	2 (13.2)	4 (20.0)	35 (15.4)	6 (18.2)	6 (24.0)	29 (51.7)
	Moderately differentiated	100 (38.0)	5 (26.3)	2 (28.6)	93 (39.2)	7 (46.8)	8 (40.0)	85 (37.3)	12 (36.4)	9 (36.0)	12 (21.4)
	Poorly differentiated	101 (38.4)	10 (52.6)	1 (14.2)	90 (38.0)	6 (40.0)	5 (25.0)	90 (39.5)	15 (45.4)	6 (24.0)	15 (15.9)
	Missing	41 (15.6)	0 (0.0)	2 (28.6)	19 (8.0)	0 (0.0)	3 (15.0)	18 (7.8)	0 (0.0)	4 (16.0)	0 (0.0)
	*p*-value *		0.149			0.663			0.232		
Stage											
	0	23 (8.8)	0 (0.0)	0 (0.0)	23 (9.7)	0 (0.0)	4 (20.0)	19 (8.3)	0 (0.0)	4 (16.0	19 (9.3)
	I	104 (39.5)	9 (47.4)	2 (28.6)	93 (39.2)	8 (53.4)	6 (30.0)	90 (39.5)	16 (48.5)	7 (28.0)	81 (39.5)
	II	65 (24.7)	6 (31.6)	3 (42.8)	56 (23.6)	3 (20.0)	5 (25.0)	57 (25.0)	9 (27.2)	7 (28.0)	49 (23.9)
	III	28 (10.7)	1 (5.2)	0 (0.0)	27 (11.4)	2 (13.3)	1 (5.0)	25 (11.0)	3 (9.1)	1 (4.0)	24 (11.7)
	IV	8 (3.0)	0 (0.0)	0 (0.0)	8 (3.4)	2 (13.3)	0 (0.0)	6 (2.6)	2 (6.1)	0 (0.0)	6 (2.9)
	Missing	35 (13.3)	3 (15.8)	2 (28.6)	30 (12.7)	0 (0.0)	4 (20.0)	31 (13.6)	3 (9.1)	6 (24.0)	26 (12.7)
	*p*-value *		0.619			0.134			0.288		
Tumor invasiveness											
	In situ	31 (11.8)	0 (0.0)	2 (28.6)	29 (12.2)	0 (0.0)	6 (30.0)	25 (11.0)	0 (0.0)	8 (32.0)	23 (11.2)
	Invasive	232 (88.2)	19 (100.0)	5 (71.4)	208 (87.8)	15 (100.0)	14 (70.0)	203 (89.0)	33 (100.0)	17 (68.0)	182 (88.8)
	*p*-value		0.106			**0.014**			**0.001**		
Ki67											
	High (≥14)	146 (55.5)	15 (78.9)	3 (42.8)	128 (54.0)	13 (86.6)	10 (40.0)	123 (53.9)	27 (81.8)	11 (44.0)	108 (52.7)
	Low (<14)	56 (22.3)	0 (0.0)	2 (28.6)	54 (22.8)	1 (6.7)	3 (15.0)	52 (22.8)	1 (3.0)	5 (20.0)	50 (24.4)
	Missing	61 (23.2)	4 (21.1)	2 (28.6)	55 (23.2)	1 (6.7)	7 (35.0)	53 (23.3)	5 (15.2)	9 (36.0)	47 (22.9)
	*p*-value *		0.149			0.094			**0.008**		
St. Gallen subtype											
	Luminal A	78 (29.7)	5 (26.3)	2 (28.5)	71 (30.0)	11 (73.3)	7 (35.0)	60 (26.3)	15 (45.5)	7 (28.0)	56 (27.3)
	Luminal B	46 (17.5)	0 (0.0)	1 (14.3)	45 (19.0)	1 (6.7)	2 (10.0)	43 (18.8)	1 (3.0)	3 (12.0)	42 (20.6)
	HER2 +	13 (4.9)	0 (0.0)	0 (0.0)	13 (5.5)	0 (0.0)	0 (0.0)	13 (5.7)	0 (0.0)	0 (0.0)	13 (6.3)
	Triple negative	38 (14.4)	10 (52.6)	1 (14.3)	27 (11.3)	2 (13.3)	3 (15.0)	33 (14.5)	12 (36.4)	4 (16.0)	22 (10.7)
	Missing	88 (33.5)	4 (21.1)	3 (42.9)	81 (34.2)	1 (6.7)	8 (40.0)	79 (34.7)	5 (15.1)	11 (44.0)	72 (35.1)
	*p*-value *		**0.001**			**0.02**			**<0.001**		
Exitus											
	Living	250 (95.1)	17 (89.5)	7 (100.0)	226 (95.3)	14 (93.3)	18 (90.0)	218 (95.6)	30 (90.9)	23 92.0)	197 (96.1)
	Dead	13 (4.9)	2 (10.5)	0 (0.0)	11 (4.6)	1 (6.7)	2 (10.0)	10 (4.4)	3 (9.1)	2 (8.0)	8 (3.9)
	*p*-value *		0.434			0.513			0.382		

Abbreviations: VUS, Variant of Uncertain Significance; HER2, Human Epidermal Growth Factor Receptor 2. * Pearson Chi-square test or the Fisher Exact test, as appropriate. ** One patients possess the pathogenic variants of the both *BRCA1* and *BRCA2* genes simultaneously (ID 606). *** the individuals were all Caucasians.
